# Erector spinae plane block for opioid sparing in children undergoing laparoscopic appendectomy: a randomized controlled trial

**DOI:** 10.3389/fped.2026.1803495

**Published:** 2026-05-01

**Authors:** Ming-wen Yang, Yu-zhu Cai, Ling-li Zhang, Jun Wang, Ran Tang, Ying-ying Sun

**Affiliations:** 1 Department of Anesthesiology, Anhui Provincial Children's Hospital, Hefei, Anhui, China; 2 Department of Pediatric General Surgery, Anhui Provincial Children's Hospital, Hefei, Anhui, China

**Keywords:** children, erector spinae plane block, laparoscopic appendectomy, opioid-sparing effects, postoperative pain

## Abstract

**Background:**

Despite being a minimally invasive procedure, laparoscopic appendectomy (LA) frequently induces substantial postoperative pain in children. While erector spinae plane block (ESPB) has demonstrated efficacy for postoperative analgesia in pediatric open abdominal surgery, its analgesic benefits and safety profile in laparoscopic procedures remain unestablished.

**Purpose:**

To evaluate the opioid-sparing effects, analgesic efficacy, and safety of ESPB in children undergoing LA.

**Design:**

A single-center, double-blind, randomized, superiority trial.

**Methods:**

Children aged 6–12 years with American Society of Anesthesiologists (ASA) physical status I–II scheduled for LA at Anhui Provincial Children's Hospital were enrolled. Participants were randomly allocated 1:1 using a computer-generated sequence to receive either bilateral ultrasound-guided ESPB at T8 (0.25% ropivacaine, 0.5 mL/kg per side) after tracheal intubation (ESPB group) or no block (Control group). Both groups received standardized multimodal analgesia comprising hydromorphone-based patient-controlled intravenous analgesia (PCIA) and scheduled acetaminophen. The primary outcome was 0–24 h cumulative hydromorphone consumption; secondary outcomes included pain scores, PCIA parameters, rescue analgesia requirements, recovery milestones, parental satisfaction, and adverse events.

**Results:**

Of the 80 children randomized (40 per group), 75 completed follow-up and were analyzed (ESPB, *n* = 37; control, *n* = 38). The ESPB group exhibited significantly lower 24 h hydromorphone consumption (32.8 ± 10.1 vs. 72.9 ± 14.5 μg/kg; mean difference: −40.1 μg/kg; *P* < 0.001), representing a 55% reduction compared with the Control group. Secondary outcomes favoring ESPB included lower pain scores during the early postoperative period (PACU to 6 h; *P* < 0.05), prolonged time to first PCIA demand (201.0 vs. 58.5 min; *P* < 0.001), fewer total PCIA presses (10 vs. 17; *P* < 0.001) and effective presses (9 vs. 17; *P* < 0.001) within 0–24 h, reduced rescue analgesia requirements (2.7% vs. 21.1%; *P* = 0.028), and higher parental satisfaction scores (8 vs. 7 points; *P* = 0.001). No serious block-related complications occurred.

**Conclusions:**

In children undergoing LA, a single-injection bilateral ultrasound-guided ESPB at T8 provides significant opioid-sparing effects and alleviates acute postoperative pain during the first 24 h without increasing adverse event rates, supporting its incorporation as a component of multimodal analgesia for postoperative pain management in this population.

**Clinical Trial Registration**: https://www.chictr.org.cn, identifier ChiCTR2500108148, Date of Registration: August 26, 2025.

## Introduction

Acute appendicitis represents the most common cause of emergency abdominal surgery worldwide, with an estimated lifetime risk of 6.7%–8.6% in the general population and a peak incidence during childhood and adolescence (1). Laparoscopic appendectomy (LA) was first performed by Kurt Semm in the early 1980s and has since gained precedence over the open approach due to benefits including reduced wound complications and shorter hospital stays (2, 3). Nevertheless, postoperative pain in children following LA is frequently underestimated and inadequately managed (4). Clinical surveys indicate that despite multimodal analgesia involving acetaminophen, non-steroidal anti-inflammatory drugs (NSAIDs) and opioids, over 33% of children still experience persistent moderate-to-severe pain (pain score ≥ 4, persisting for more than 60% of the assessment period) on the day of LA surgery (5).

Regional anesthesia, such as local anesthetic wound infiltration, intraperitoneal local anesthetic spray, and transvs. abdominis plane (TAP) block, is also used as part of multimodal analgesia to manage postoperative pain in children after LA (6–8). However, based on current clinical evidence, its analgesic effect is limited (4, 6–8).

The erector spinae plane block (ESPB) was first reported by Forero et al. (9) in 2016, initially for the management of thoracic neuralgia and post-thoracic surgery pain. Anatomical studies of ESPB have demonstrated its potential spread to the paravertebral space and epidural space; thus, when administered at the corresponding vertebral levels (T7, T8), it can exert extensive analgesic effects on both abdominal somatic and visceral pain, leading to growing attention for its application in abdominal surgeries (10, 11). Several randomized controlled trials (RCTs) have verified the efficacy of ESPB in pediatric open abdominal surgeries (11–13); however, its clinical application in pediatric laparoscopic surgeries remains scarce to date. To address this research gap, we designed this RCT. We hypothesized that ultrasound-guided bilateral ESPB at T8 in children undergoing LA, compared with systemic analgesic regimens, would reduce postoperative 24 h opioid consumption (primary outcome) and alleviate postoperative pain. Additionally, we evaluated its clinical safety profile.

## 2 Materials and methods

### 2.1 Trial design

This study was conducted as a double-blind, randomized controlled clinical trial at a Grade A tertiary children's hospital located in an eastern Chinese province and was reported in accordance with the Consolidated Standards of Reporting Trials (CONSORT) guidelines. The study protocol, which complied with the ethical requirements of the Declaration of Helsinki for human research, received approval from the Medical Research Ethics Committee of Anhui Provincial Children's Hospital (Approval No: EYLL-2025-035) and was prospectively registered with the Chinese Clinical Trial Registry (Website: http://www.chictr.org.cn; Registration No: ChiCTR2500108148; Date of registration: August 26, 2025). Patient recruitment occurred from September 1, 2025 (first patient enrolled date) to December 31, 2025 (last patient enrolled date). Before enrollment, the legal guardians of all children were fully informed of the trial protocol and signed written informed consent.

### 2.2 Participants

Inclusion criteria were children aged 6–12 years, with a body mass index (BMI) between the 5th and 95th percentiles for age and sex according to World Health Organization (WHO) growth standards (14), classified as American Society of Anesthesiologists (ASA) physical status I or II, clinically diagnosed with suspected acute appendicitis, and scheduled for LA within 24 h. Exclusion criteria comprised known allergy to local anesthetics or acetaminophen, infection at the puncture site, coagulopathy, thoracolumbar spinal anatomical deformities (e.g., scoliosis) or neurological disorders (e.g., tethered cord, epilepsy, cognitive impairment), cardiopulmonary diseases (e.g., congenital heart disease, respiratory failure), liver or kidney dysfunction, chronic use of opioids or NSAIDs (>3 months), inability of guardians to demonstrate competency in patient-controlled intravenous analgesia (PCIA) operation after standardized training, or refusal of informed consent by guardians. Additionally, patients with cancellation or postponement of surgery after randomization, unsuccessful ESPB implementation, or withdrawal of informed consent by guardians were excluded from the final data analysis.

Prior to randomization, both children and their guardians underwent a standardized training program lasting approximately 20 min, conducted by Acute Pain Service (APS) nurses. Guardians received structured hands-on instruction covering: (1) age-appropriate application of pain assessment tools, specifically the Face, Legs, Activity, Cry, Consolability (FLACC) scale for children aged <8 years and the Numeric Rating Scale (NRS) for those aged ≥ 8 years (15, 16), alongside the predefined pain threshold (score ≥ 4); (2) stepwise operation of the PCIA pump (bolus activation, lockout interval recognition, and alarm response); and (3) safety protocols (contraindications for administration during sleep or sedation, maximum allowable frequency, and escalation procedures to clinical staff). Competency was evaluated using an institutionally developed 8-item direct-observation checklist (Supplementary Materials S1), with guardians deemed qualified only upon achieving full competency on this checklist. Consistent with the study protocol, randomization was permitted exclusively for children with at least one primary guardian who achieved full competency on this checklist.

### 2.3 Randomization and blinding

An independent statistician generated the allocation sequence using SPSS software (version 22.0; IBM Corp, Armonk, NY, USA) via a stratified block randomization method with a fixed block size of 4. Eligible patients were randomly assigned to either the ESPB group or the Control group at a 1:1 ratio. Stratification was based on preoperative clinical and imaging assessment of appendicitis severity: (1) uncomplicated appendicitis (localized right lower quadrant tenderness without signs of diffuse peritonitis, and ultrasound showing appendiceal diameter >6 mm without significant free fluid or abscess); (2) complicated appendicitis (clinical signs of diffuse peritonitis or imaging evidence of free fluid >10 mL, abscess, or suspected perforation on preoperative ultrasound/CT) (17). Allocation details were concealed in sequentially numbered, opaque, sealed envelopes prepared by an independent research coordinator; envelopes were opened by a circulating nurse immediately before anesthesia induction. The preparation of 0.25% ropivacaine and performance of ESPB were conducted exclusively in the ESPB group by a designated anesthesiologist with extensive experience in nerve blocks; the Control group received no injection but had a sham dressing applied at the T8 level. The statistician and the anesthesiologist who performed the nerve blocks did not participate in subsequent patient management or outcome assessment. Throughout the trial, the children, their guardians, intraoperative attending anesthesiologists, operating surgeons, postoperative outcome assessors, and all other healthcare providers involved in their care remained blinded to group assignment.

### 2.4 Anesthesia, surgery, and analgesia techniques

Standard intraoperative monitoring included heart rate (HR), non-invasive blood pressure (NIBP), peripheral oxygen saturation (SpO₂), and bispectral index (BIS). General anesthesia was induced with intravenous administration of sufentanil (0.3 μg/kg), cisatracurium (0.15 mg/kg), and propofol (3 mg/kg). After endotracheal intubation, mechanical ventilation was adjusted to maintain end-tidal carbon dioxide (EtCO₂) at 35–45 mmHg. Anesthesia was maintained with continuous infusion of propofol (4–8 mg/kg/h) and remifentanil (0.1–0.3 μg/kg/min). Lactated Ringer's solution was administered at 10 mL/kg/h. Doses of intravenous anesthetics were titrated to maintain a BIS value of 40–60 and HR/NIBP fluctuations within ±20% of baseline values. At the end of surgery, all intravenous anesthetics were discontinued. After extubation, patients were transferred to the post-anesthesia care unit (PACU) for continuous monitoring and were returned to the ward upon achieving an Aldrete score ≥ 9 (18) and a pain score < 4.

All LA procedures were consistently performed by a single surgeon using a standardized single-incision laparoscopic appendectomy technique (19).

All children received a standardized multimodal analgesia regimen. Upon admission, acetaminophen 15 mg/kg was administered orally or intravenously to patients who had not received acetaminophen or NSAIDs within 6 h prior to admission. At the end of surgery, ondansetron 0.1 mg/kg was administered intravenously, followed by connection to the PCIA pump. The pump was prepared with hydromorphone 150 μg/kg diluted in normal saline to a total volume of 100 mL and programmed in bolus-only mode (no basal infusion), with a single PCIA bolus volume of 2 mL (equivalent to hydromorphone 3 μg/kg) and a 20 min lockout interval. Guardians were authorized to activate bolus doses when the child's pain score was ≥4, based on their training in pain behavior recognition. Postoperatively, acetaminophen 15 mg/kg was administered every 8 h for 48 h. If pain remained uncontrolled (score ≥ 4 at 20 min post-bolus), the APS (blinded to group assignment) was consulted for rescue analgesia with hydromorphone 10 μg/kg administered intravenously over 2–3 min. The APS physician independently determined whether to initiate a supplementary basal infusion (1 mL/h) after excluding surgical complications. The hydromorphone dosing strategy—including PCIA bolus (3 μg/kg), rescue dose (10 μg/kg), and potential basal infusion (1.5 μg/kg/h)—was designed to remain within the established safety range for children (20).

### 2.5 Intervention

General anesthesia induction and all nerve blocks were performed in a dedicated induction room. Following endotracheal intubation, patients allocated to the ESPB group were placed in the prone position to receive the ESPB (21). The T8 vertebral spinous process was identified through manual palpation, counting caudally from C7 and cranially from T12. A high-frequency linear-array transducer (L14-5wu; Mindray, China) was positioned longitudinally approximately 2–3 cm lateral to the midline. Under real-time ultrasonography, the erector spinae muscle, T8 transverse process, and pleural line were clearly identified. Using an in-plane approach, a 20-gauge needle (AN-N; Yaguang Corporate, China) was advanced from caudal to cephalad until the tip was satisfactorily positioned deep to the erector spinae muscle and superficial to the transverse process. After negative aspiration for blood, a test dose of 1 mL normal saline was injected to confirm adequate hydrodissection. This was followed by administration of 0.25% ropivacaine at 0.5 mL/kg (maximum 20 mL per side). The identical procedure was subsequently performed on the contralateral side. Patients randomized to the Control group did not receive any nerve block. All patients were then transferred to the operating room for surgery.

### 2.6 Outcome measurements

Follow-up was conducted by designated postoperative follow-up staff blinded to group assignment at 48 h postoperatively, and data were collected through the electronic medical record system, PCIA pump electronic records, and interviews with patients and their guardians.

Baseline characteristics collected included age, sex, BMI, ASA physical status classification, presence of fever (axillary temperature > 37.5 °C), and appendicitis type (complicated or uncomplicated) (17). Intraoperative data included doses of remifentanil, propofol, and lactated Ringer's solution; duration of anesthesia (defined as the interval from initiation of anesthesia induction to tracheal extubation); time to extubation (defined as the interval from the end of surgery to tracheal extubation); and duration of surgery (defined as the interval from skin incision to completion of skin closure). PACU stay duration and postoperative hospital length of stay were also recorded.

The primary outcome was the total cumulative dose of hydromorphone (μg/kg) administered during the first 0–24 h after surgery, calculated as the sum of hydromorphone delivered by the PCIA pump and rescue analgesia doses. The 24–48 h cumulative hydromorphone consumption was prespecified as a secondary outcome.

Secondary outcomes included the following: (1) preoperative pain scores, the highest pain score in the PACU, and pain scores at rest and during active movement (turning in bed or deep breathing) at 3, 6, 12, 24, and 48 h postoperatively; pain assessment was conducted by specially trained PACU and ward nurses blinded to group assignment, using the FLACC scale for children <8 years and the NRS for those ≥8 years (15, 16). Pain scores were assessed at predetermined time points and sleeping patients without pain behaviors were assigned a score of 0. (2) Time to first PCIA demand. (3) Total and effective PCIA button presses during the 0–24 h and 24–48 h postoperative periods. (4) Rescue analgesia rate within 48 h (proportion of patients requesting additional analgesia from the APS). (5) Time to first ambulation. (6) Time to first flatus. (7) Time to first oral intake. (8) Parental satisfaction with analgesia (rated on a 0–10 scale, where 0 = complete dissatisfaction and 10 = complete satisfaction). All time-to-event outcomes were calculated from the end of surgery.

Puncture-related adverse events included: (1) pneumothorax (confirmed by intraoperative or postoperative chest X-ray); (2) subcutaneous hematoma or bleeding at the puncture site (visible ecchymosis > 2 cm in diameter or active bleeding requiring pressure for > 2 min). Opioid-related adverse events included: (1) postoperative nausea and vomiting (PONV), defined as absent (no nausea, retching, or vomiting) or present (occurrence of any of nausea, retching, or vomiting); (2) pruritus (patient-reported itching with or without scratching behavior); (3) respiratory depression (respiratory rate < 10 breaths/min for >1 min) or hypoxemia (SpO₂ < 90% for >1 min despite supplemental oxygen at 2 L/min via nasal cannula).

### 2.7 Sample size calculation

Sample size calculation was based on the preset primary outcome of the trial. According to a pilot study with 10 cases, the hydromorphone dose within the first 24 h after pediatric LA was 75 ± 26.9 μg/kg. We anticipated that the implementation of ESPB would reduce the opioid dose in children within the first 24 h after surgery by at least 30% (22). Using PASS software (version 15.0; NCSS LLC, Kaysville, UT, USA), with a significance level of 0.05 and a test power of 0.9, the calculation showed that 32 children were needed in each group. Considering a 20% dropout rate, 40 children were needed in each group.

### 2.8 Statistical analysis

Statistical analyses were performed using IBM SPSS Statistics (version 22.0; IBM Corp, Armonk, NY, USA) and GraphPad Prism (version 9.0; GraphPad Software, San Diego, CA, USA). Based on the Shapiro–Wilk test, normally distributed continuous variables were presented as mean ± standard deviation (SD) and compared between groups using the independent samples *t*-test; between-group mean differences and their 95% confidence intervals (CIs) were calculated. Non-normally distributed data were presented as median [interquartile range (IQR)] and compared using the Mann–Whitney *U* test; the Hodges–Lehmann estimator was used to determine the median difference with its 95% CI. Categorical variables were expressed as frequency (%) and analyzed using the *χ²* test or Fisher's exact test (for expected cell counts < 5); risk ratios (RRs) with 95% CIs were calculated. *P* < 0.05 was considered to indicate a statistically significant difference.

A generalized estimating equations (GEE) model with an exchangeable working correlation matrix and a linear link function was applied to analyze the repeated measurement data of pain scores, both at rest and during active movement, across the predefined follow-up time points. Treatment group (ESPB group vs. Control group) and measurement time point were set as the main effects in the model. Meanwhile, given the age-appropriate pain assessment tools used in this study (FLACC scale for children < 8 years old, and NRS for children ≥ 8 years old), age group was incorporated into the model as a covariate to adjust for the potential systematic bias caused by age-stratified pain assessment. The group × time interaction term, group × age group interaction term, and group × time × age group three-way interaction term were also included in the model simultaneously. *post-hoc* pairwise comparisons between groups at each time point were performed with the Bonferroni method for multiple testing correction.

Exploratory subgroup analyses were carried out based on the type of appendicitis (complicated vs. uncomplicated) for the primary outcome (0–24 h cumulative hydromorphone consumption). Between-group comparisons within each subgroup were performed using the independent samples *t*-test. To evaluate the consistency of ESPB intervention effect across subgroups and quantify the between-subgroup difference in intervention effect, a linear regression model was constructed. In this model, the dependent variable was the 0–24 h cumulative hydromorphone consumption, and the independent variables included ESPB intervention, the type of appendicitis, and their interaction term.

## 3 Results

### 3.1 Participant flow

**Figure 1:**
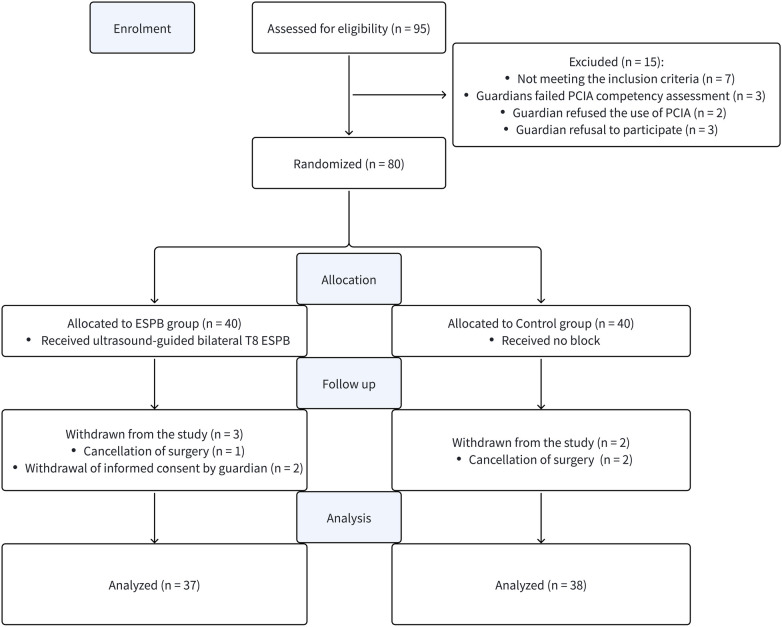
CONSORT flow diagram of this randomized controlled trial. ESPB, erector spinae plane block; PCIA, patient-controlled intravenous analgesia.

Initially, 95 children were screened for eligibility in this study. Of these, 12 were excluded prior to competency assessment: 7 for failing to meet inclusion criteria, 2 due to guardian refusal of PCIA use, and 3 due to refusal to participate. The remaining 83 eligible patients (comprising 86 designated guardians) underwent the standardized PCIA training and competency assessment. Among the 86 guardians assessed, 80 successfully passed, yielding a guardian-level passing rate of 93.0%. Three patients were excluded because both of their participating guardians failed the competency evaluation. Consequently, the remaining 80 patients, each accompanied by at least one guardian who had successfully passed the competency assessment, were randomized in a 1:1 ratio to the ESPB group (*n* = 40) or the Control group (*n* = 40). During the subsequent study period, 3 patients were withdrawn due to surgical cancellation and 2 due to withdrawal of informed consent by guardians. Ultimately, 75 patients (ESPB group, *n* = 37; Control group, *n* = 38) completed postoperative follow-up and were included in the final analysis. All patients in the ESPB group underwent successful ESPB implementation. The detailed study flow in accordance with the CONSORT guidelines is presented in Figure 1.

### 3.2 Baseline and intraoperative characteristics

Baseline characteristics are presented in Table 1. The ESPB group and Control group were well balanced with respect to age, sex, BMI, ASA physical status, fever status, and appendicitis classification.

**Table 1:** Baseline and intraoperative characteristics of children undergoing LA in the two groups.

Variables	ESPB group (*n* = 37)	Control group (*n* = 38)	*P* value
Age (years)	9 (7, 9.5)	8 (7, 10)	0.935
<8 years	11 (29.7)	12 (31.6)	0.862
8–12 years	26 (70.3)	26 (68.4)	
Sex (*n*%)			0.202
Male	15 (40.5)	21 (55.3)	
Female	22 (59.5)	17 (44.7)	
BMI (kg/m^2^)	22.7 (19.2, 25.4)	23.6 (20.0, 24.9)	0.840
ASA class (*n*%)			0.160
I	28 (75.7)	23 (60.5)	
II	9 (24.3)	15 (39.5)	
Fever (*n*%)	24 (64.9)	27 (71.1)	0.566
Classification of Acute Appendicitis (*n*%)			0.572
Uncomplicated Acute Appendicitis	16 (43.2)	14 (36.8)	
Complicated Acute Appendicitis	21 (56.8)	24 (63.2)	
Intraoperative remifentanil dose (μg/kg)	5.8 ± 1.7	6.3 ± 1.6	0.240
Intraoperative propofol dose (mg/kg)	4.9 ± 0.6	5.1 ± 0.5	0.067
Intraoperative lactated Ringer's solution dose (mL/kg)	6.9 ± 0.9	6.9 ± 0.7	0.848
Duration of surgery (min)	35.6 ± 7.2	38.1 ± 3.7	0.062
Duration of anesthesia (min)	65.7 ± 6.1	62.5 ± 9.4	0.089
Extubation time (min)	12 (10, 16)	14 (11, 17)	0.154
PACU stay duration (min)	32 (28, 40)	38 (30, 42)	0.065

Data are expressed as mean ± SD, median (IQR), or number (%).

**Interventions:** ESPB group, ultrasound-guided bilateral T8 ESPB with 0.25% ropivacaine 0.5 mL/kg per side after tracheal intubation; Control group, no block.

LA, laparoscopic appendectomy; ESPB, erector spinae plane block; BMI, body mass index; ASA, American Society of Anesthesiologists; PACU, post-anesthesia care unit.

Intraoperative characteristics are summarized in Table 1. No statistically significant differences were observed between groups in intraoperative remifentanil dose (5.8 ± 1.7 vs. 6.3 ± 1.6 μg/kg; *P* = 0.240), propofol dose (4.9 ± 0.6 vs. 5.1 ± 0.5 mg/kg; *P* = 0.067), lactated Ringer's solution administration (6.9 ± 0.9 vs. 6.9 ± 0.7 mL/kg; *P* = 0.848), duration of surgery (35.6 ± 7.2 vs. 38.1 ± 3.7 min; *P* = 0.062), duration of anesthesia (65.7 ± 6.1 vs. 62.5 ± 9.4 min; *P* = 0.089), time to extubation [12 (10, 16) vs. 14 (11, 17) min; *P* = 0.154], or PACU stay duration [32 (28, 40) vs. 38 (30, 42) min; *P* = 0.065].

### 3.3 Primary outcome

**Figure 2:**
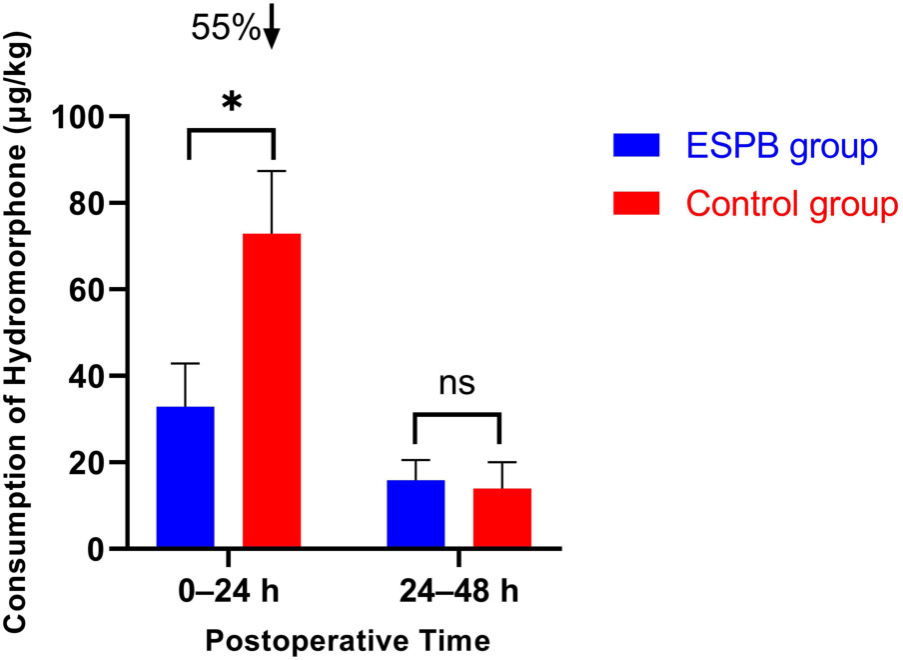
Comparison of cumulative hydromorphone consumption at 0–24 h and 24–48 h postoperatively between the two groups. Error bars represent standard deviation. *P < 0.05 vs. Control group. **Interventions:** ESPB group, ultrasound-guided bilateral T8 ESPB with 0.25% ropivacaine 0.5 mL/kg per side after tracheal intubation; Control group, no block. ESPB, erector spinae plane block; ns, not significant.

The primary outcome, total cumulative hydromorphone consumption within 0–24 h postoperatively, is presented in Table 2 and Figure 2. The ESPB group demonstrated a significant reduction in hydromorphone consumption compared to the Control group, with a mean difference of −40.1 μg/kg (95% CI: −45.8 to −34.3; *P* < 0.001). The cumulative dose in the ESPB group (32.8 ± 10.1 μg/kg) was 55% lower than in the Control group (72.9 ± 14.5 μg/kg).

**Table 2:** Comparison of primary and secondary postoperative outcomes in children undergoing LA between the two groups.

Variables	ESPB group (*n* = 37)	Control group (*n* = 38)	MD/RR (95% CI)	*P* value
The total cumulative dose of hydromorphone within 0–24 h postoperatively (μg/kg)^#^	32.8 ± 10.1	72.9 ± 14.5	−40.1 (−45.8 to −34.3)	<0.001*
The total cumulative dose of hydromorphone within 24–48 h postoperatively (μg/kg)	15.6 ± 4.9	15.5 ± 5.0	0.1 (−2.2 to 2.4)	0.936
Postoperative time to first PCIA bolus press (min)	201.0 (156.0, 306.0)	58.5 (50.0, 69.0)	143.0 (128.0 to 161.0)	<0.001*
Total number of PCIA bolus presses within 0–24 h postoperatively (times)	10 (8, 11)	17 (15, 19)	−7 (−8 to −6)	<0.001*
Effective number of PCIA bolus presses within 0–24 h postoperatively (times)	9 (8, 11)	17 (14, 19)	−7 (−8 to −6)	<0.001*
Total number of PCIA bolus presses within 24–48 h postoperatively (times)	5 (2, 11)	5 (4, 6)	0 (−1 to 4)	0.521
Effective number of PCIA bolus presses within 24–48 h postoperatively (times)	5 (2, 9)	5 (4, 6)	0 (−1 to 2)	0.572
Rescue analgesia rate within 48 h postoperatively (%)	1 (2.7)	8 (21.1)	0.13 (0.02 to 0.98)	0.028*
Postoperative time to first ambulation (h)	7 (6, 9)	8 (6, 10)	−1 (−1 to 0)	0.105
Postoperative time to first flatus (h)	26 (23, 31)	28 (26, 30)	−2 (−4 to 0)	0.076
Postoperative time to first oral intake (h)	29 (24, 36)	31 (29, 35)	−2 (−5 to 1)	0.143
Postoperative length of stay (d)	4 (3, 5)	4 (3, 5)	0 (−1 to 1)	0.991
Parental satisfaction with analgesia (0–10 Points)	8 (7, 9)	7 (6, 8)	1 (1 to 2)	0.001*

Data are expressed as mean ± SD, median (IQR), MD (95% CI), or RR (95% CI).

**Interventions:** ESPB group, ultrasound-guided bilateral T8 ESPB with 0.25% ropivacaine 0.5 mL/kg per side after tracheal intubation; Control group, no block.

LA, laparoscopic appendectomy; ESPB, erector spinae plane block; MD, median/mean difference; RR, risk ratio; CI, confidence interval; PCIA, patient-controlled intravenous analgesia.

* *P* < 0.05 vs. Control group.

# Indicates primary outcome.

In contrast, no significant difference was observed in the 24–48 h period, with similar hydromorphone consumption between groups (ESPB group: 15.6 ± 4.9 μg/kg vs. Control group: 15.5 ± 5.0 μg/kg; mean difference: 0.1 μg/kg, 95% CI: −2.2 to 2.4; *P* = 0.936).

### 3.4 Secondary outcomes

**Figure 3:**
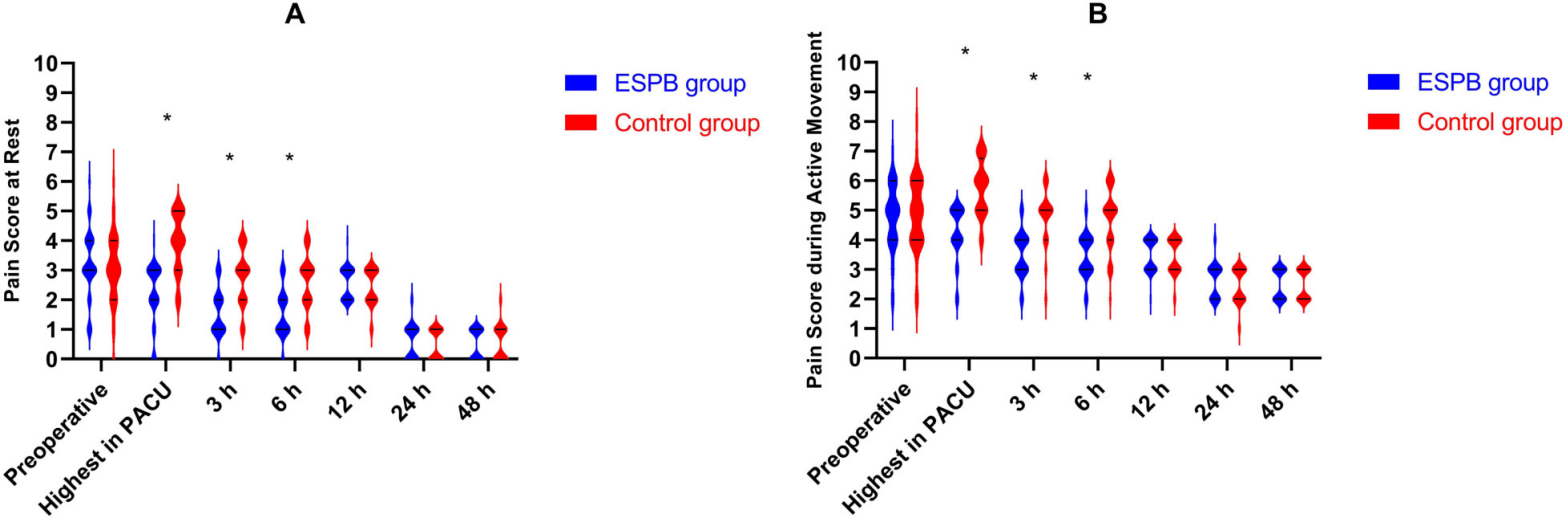
Comparison of perioperative pain scores from preoperative to 48 h postoperatively between the two groups. **(A)** Pain scores at rest; **(B)** Pain scores during active movement. The black line represents the upper and lower quartiles. *P < 0.05 vs. Control group (Bonferroni-corrected). **Interventions:** ESPB group, ultrasound-guided bilateral T8 ESPB with 0.25% ropivacaine 0.5 mL/kg per side after tracheal intubation; Control group, no block. ESPB, erector spinae plane block; PACU, post-anesthesia care unit.

Perioperative pain scores at rest and during active movement, analyzed by the GEE model adjusted for age group as a covariate, are presented in Figure 3. During the PACU period, the ESPB group demonstrated significantly lower pain scores compared to the Control group both at rest (2.22 vs. 3.92; *P* < 0.001) and during movement (4.22 vs. 5.76; *P* < 0.001). At 3 h postoperatively, significant reductions persisted at rest (1.54 vs. 2.79; *P* = 0.014) and during movement (3.52 vs. 4.71; *P* = 0.007). Similarly, at 6 h postoperatively, the ESPB group showed significantly lower pain scores at rest (1.57 vs. 2.61; *P* = 0.002) and during movement (3.46 vs. 4.66; *P* = 0.002). No statistically significant differences were observed between groups at preoperative, 12, 24, or 48 h postoperatively for either rest or movement pain scores (all *P* > 0.05).

GEE analysis adjusted for age group as a covariate revealed three key findings: (1) the significant group main effect (*P* < 0.001) indicates that overall, patients in the ESPB group had lower pain scores than those in the Control group throughout the perioperative period; (2) the significant time main effect (*P* < 0.001) confirms that pain scores varied across different time points in the perioperative trajectory; and (3) the significant group × time interaction (*P* < 0.001) demonstrates that the pattern of pain score changes over time differed between groups. Notably, the group × age group interaction and the group × time × age group three-way interaction were both nonsignificant (*P* > 0.05 for both), indicating that the analgesic effect of ESPB was consistent across different age groups and that age did not modify the temporal trend of pain scores between groups. The complete GEE model results, including Wald *χ²* statistics, estimated marginal means, and pairwise comparisons, are presented in Supplementary Materials S2.

Secondary outcomes are presented in Table 2. The ESPB group demonstrated significantly prolonged time to first PCIA bolus press compared to the Control group [201.0 (156.0, 306.0) vs. 58.5 (50.0, 69.0) minutes; median difference: 143.0 min; 95% CI: 128.0–161.0; *P* < 0.001]. The total number of PCIA presses within 0–24 h was significantly lower in the ESPB group [10 [8, 11] vs. 17 [15, 19] presses; median difference: −7; 95% CI: −8 to −6; *P* < 0.001], as was the effective number of presses [9 (8, 11) vs. 17 (14, 19) presses; median difference: −7; 95% CI: −8 to −6; *P* < 0.001]. No significant differences were observed in the 24–48 h period for either total PCIA presses [5 (2, 11) vs. 5 (4, 6); *P* = 0.521] or effective presses [5 (2, 9) vs. 5 (4, 6); *P* = 0.572].

The rescue analgesia rate within 48 h was lower in the ESPB group (2.7% vs. 21.1%; RR: 0.13; 95% CI: 0.02–0.98; *P* = 0.028). No statistically significant differences were observed between groups in time to first ambulation [7 (6, 9) vs. 8 (6, 10) hours; *P* = 0.105], time to first flatus [26 (23, 31) vs. 28 (26, 30) hours; *P* = 0.076], or time to first oral intake [29 (24, 36) vs. 31 (29, 35) hours; *P* = 0.143].

Postoperative hospital length of stay was similar between groups [4 (3, 5) days in both groups; *P* = 0.991]. Parental satisfaction with analgesia was significantly higher in the ESPB group [8 (7, 9) vs. 7 (6, 8) points; median difference: 1 point; 95% CI: 1–2; *P* = 0.001].

### 3.5 Adverse events

The incidence of adverse events is presented in Table 3. No pneumothorax or respiratory depression/hypoxemia occurred in either group. Two patients (5.4%) in the ESPB group experienced hematoma or bleeding at the puncture site compared with none in the Control group. PONV occurred in 5 patients (13.5%) in the ESPB group vs. 9 patients (23.7%) in the Control group (RR: 0.57; 95% CI: 0.21–1.54; *P* = 0.258). Pruritus was reported in 2 patients (5.4%) in the ESPB group and 4 patients (10.5%) in the Control group (RR: 0.51; 95% CI: 0.10–2.64; *P* = 0.674). No statistically significant differences were observed between groups in the incidence of any adverse event.

**Table 3:** Comparison of the incidence of adverse events within 48 h in children undergoing LA between the two groups.

Adverse events	ESPB group (*n* = 37)	Control group (*n* = 38)	RR (95% CI)	*P* value
Pneumothorax (%)	0 (0)	0 (0)	NA	NA
Hematoma or bleeding (%)	2 (5.4)	0 (0)	NA*	0.240
PONV (%)	5 (13.5)	9 (23.7)	0.57 (0.21 to 1.54)	0.258
Pruritus (%)	2 (5.4)	4 (10.5)	0.51 (0.10 to 2.64)	0.674
Respiratory depression or hypoxemia (%)	0 (0)	0 (0)	NA	NA

LA, laparoscopic appendectomy; Data are expressed as number (%), RR (95% CI).

**Interventions:** ESPB group, ultrasound-guided bilateral T8 ESPB with 0.25% ropivacaine 0.5 mL/kg per side after tracheal intubation; Control group, no block.

ESPB, erector spinae plane block; RR, risk ratio; CI, confidence interval; NA, not applicable; PONV, postoperative nausea and vomiting.

* RR (95% CI) not calculable due to zero event incidence in the Control group.

### 3.6 Exploratory subgroup analyses

Subgroup analysis results are presented in Table 4. Within the uncomplicated appendicitis subgroup (*n* = 30), the ESPB group demonstrated significantly lower 0–24 h hydromorphone consumption compared to the Control group (26.2 ± 10.2 vs. 59.4 ± 9.9 μg/kg; mean difference: −33.1 μg/kg; 95% CI: −40.7 to −25.6; *P* < 0.001). Similarly, within the complicated appendicitis subgroup (*n* = 45), the ESPB group showed significantly lower consumption than the Control group (37.9 ± 6.5 vs. 80.8 ± 10.3 μg/kg; mean difference: −42.9 μg/kg; 95% CI: −48.2 to −37.6; *P* < 0.001).

**Table 4:** Subgroup analysis of the primary outcome (0–24 h postoperative cumulative hydromorphone consumption) stratified by appendicitis type in children undergoing LA between the two groups.

Subgroup	Intervention Group	*n*	Primary outcome (μg/kg)	MD (95% CI)	*P* value
Uncomplicated appendicitis (*n* = 30)	ESPB group	16	26.2 ± 10.2	−33.1 (−40.7 to −25.6)	<0.001*
	Control group	14	59.4 ± 9.9		
Complicated appendicitis (*n* = 45)	ESPB group	21	37.9 ± 6.5	−42.9 (−48.2 to −37.6)	<0.001*
	Control group	24	80.8 ± 10.3		

Data are expressed as mean ± SD, number, or MD (95% CI).

**Interventions:** ESPB group, ultrasound-guided bilateral T8 ESPB with 0.25% ropivacaine 0.5 mL/kg per side after tracheal intubation; Control group, no block.

LA, laparoscopic appendectomy; ESPB, erector spinae plane block; MD, mean difference; CI, confidence interval.

* *P* < 0.05 vs. control group.

The interaction term (ESPB × appendicitis type) in the linear regression model was statistically significant (B = −9.75; standard erro*r* = 4.38; 95% CI: −18.35 to −1.16; *P* = 0.029), indicating heterogeneous treatment effects across subgroups. Specifically, ESPB provided an additional reduction of 9.75 μg/kg in hydromorphone consumption in patients with complicated appendicitis compared to those with uncomplicated appendicitis. The model showed good explanatory power (adjusted *R²* = 0.846) and was statistically significant overall (*F* = 136.66; *P* < 0.001) (Supplementary Materials S3).

Although this subgroup analysis was prespecified, the modest sample sizes within subgroups may limit the precision of the interaction estimate; therefore, these findings should be interpreted cautiously.

## 4 Discussion

This RCT demonstrates that ultrasound-guided bilateral ESPB at the T8 level, administered after tracheal intubation, significantly reduced 24 h postoperative hydromorphone consumption by 55% compared with systemic analgesia alone in children undergoing LA. Secondary outcomes favored the ESPB group, including lower pain scores during the early postoperative period (PACU to 6 h), prolonged time to first PCIA demand, reduced rescue analgesia requirements, and higher parental satisfaction scores, without increasing complication rates. To our knowledge, this is the first RCT to investigate the efficacy of ESPB for LA in the pediatric population.

Contrary to the prevailing perception that LA is a minimally invasive procedure associated with mild postoperative pain, accumulating evidence demonstrates that LA can induce substantial pain intensity comparable to major open surgeries (4, 23). In a landmark prospective cohort study involving more than 50,000 adult surgical patients, Gerbershagen et al. (23) reported that the median pain score on the first postoperative day following LA was 5.0 (0–10 NRS), a level comparable to sternotomy and total knee arthroplasty, and notably higher than that observed after open lung resection. Despite this high pain burden, opioid utilization after LA remains substantially lower than after major thoracoabdominal procedures, suggesting that postoperative pain following LA is frequently underestimated and inadequately managed (23). This phenomenon extends to the pediatric population. Liu et al. (19) documented mean pain scores of 4.80 ± 0.16 on postoperative day 1 in children undergoing single-incision laparoscopic appendectomy. Similarly, Liu et al.'s (24) retrospective study demonstrated that despite multimodal analgesia with acetaminophen and morphine PCIA, children undergoing LA experienced high pain scores during the first 24 postoperative hours—6 (3, 9) in those without peritonitis and 6 (3, 8) in those with peritonitis. These findings are consistent with the elevated pain scores observed in our control group during the early postoperative period.

Postoperative pain following LA is primarily attributed to three sources: somatic pain from surgical incisions, visceral pain induced by pneumoperitoneum and carbon dioxide-mediated peritoneal irritation, and inflammatory pain resulting from localized and diffuse inflammation of the appendix and surrounding tissues (25–27). The complexity of this pain, which encompasses both somatic and visceral components, together with its widespread distribution across the abdominal wall and viscera, poses substantial challenges for effective analgesia (1, 25). Conventional regional techniques—such as local anesthetic wound infiltration, intraperitoneal local anesthetic spray, and TAP block—demonstrate modest efficacy in LA due to their predominant targeting of somatic abdominal wall innervation and limited coverage of visceral afferents and diffuse inflammatory pain within the peritoneal cavity (1, 4, 10, 25).

To date, clinical evidence for ESPB in pediatric laparoscopic surgery remains extremely limited. Only two case reports described the successful use of ESPB in pediatric laparoscopic cholecystectomy: Aksu et al. (28) and Karaca et al. (29), involving a total of seven children, all of whom achieved satisfactory postoperative analgesia (pain scores ≤ 3) without complications. In contrast, ESPB has been extensively studied in adult laparoscopic abdominal surgery and has demonstrated effects consistent with our findings. A meta-analysis by Sia et al. (30) demonstrated that ESPB significantly reduces postoperative opioid consumption and PONV incidence in adult laparoscopic surgery. Similarly, Oraee et al.'s (31) meta-analysis confirmed that ESPB provides superior analgesia compared to placebo or TAP block, with lower pain scores, reduced opioid requirements, decreased rescue analgesia needs, and lower PONV rates.

We propose that the opioid-sparing effect and early postoperative analgesia observed with ESPB in this study primarily result from two complementary mechanisms: cephalocaudal spread of local anesthetic within the erector spinae plane affecting multiple spinal nerve dorsal rami, and potential paravertebral spread reaching ventral rami and sympathetic trunks (10, 13, 22, 31, 32). Together, these mechanisms may enable ESPB to simultaneously attenuate both somatic pain from abdominal incisions and visceral pain from pneumoperitoneum and inflammatory processes (10, 13, 30, 31). However, the precise spread pattern of local anesthetic following ESPB and its analgesic implications remain incompletely understood and subject to ongoing debate (22, 30). Supporting paravertebral spread, Yang et al. (33) demonstrated in cadaveric studies that dye injected during ESPB spread along the erector spinae plane to involve dorsal rami and penetrated the superior costotransverse ligament to reach the paravertebral space, affecting ventral rami and sympathetic trunks. Similarly, Schwartzmann et al. (34) observed in living subjects using MRI that local anesthetic spread to the erector spinae plane, neural foramina, and intercostal spaces following ESPB, producing cutaneous analgesia on both dorsal and ventral aspects of the thorax. In contrast, three independent cadaveric studies by Ivanusic et al. (35), Harbell et al. (36), and Aponte et al. (37) reported that dye remained confined to the erector spinae plane with cephalocaudal and lateral spread affecting only dorsal rami, without evidence of penetration into the paravertebral space or involvement of ventral rami. Notably, these discrepant findings may reflect fundamental differences between cadaveric and *in vivo* conditions: negative intrapleural pressure, dynamic muscle contractions, and physicochemical properties of living tissue (e.g., higher hyaluronic acid content) may collectively facilitate local anesthetic spread in clinical practice beyond what is observed in static cadaveric models (33, 34, 36, 37).

Currently, anatomical studies on ESPB in the pediatric population are still limited. Govender et al. (38) performed ESPB in a fresh, unembalmed neonate cadaver at the T8 and T10 levels using a relatively small volume of dye (1 mL/kg) and found the dye spread to the intercostal spaces and paravertebral space. Similarly, Govender et al. (39) in two fresh, unembalmed neonate cadavers, administered ESPB at T5 (0.5 mL) and T8 (0.2 mL) levels and observed dye spread to the intercostal space, paravertebral space, and even the epidural space. It is currently believed that physiological and anatomical differences between pediatric and adult populations—such as differences in spinal curvature, greater elasticity of the pediatric spine, and lower density of ligaments and cartilaginous endplates—may influence and promote the distribution of local anesthetics (21).

Cumulative 24-hour postoperative opioid consumption in the ESPB group was 55% lower than that in the control group, substantially exceeding the 30% reduction prespecified during study design based on prior evidence. We attribute this amplified effect to two main factors. First, previous literature predominantly comprised heterogeneous surgical populations, including pediatric cardiac, orthopedic, and open abdominal procedures (22). Such operations involve more complex pain mechanisms and heightened baseline perioperative inflammation, which may partially attenuate the opioid-sparing effect of ESPB. In contrast, our study focused exclusively on pediatric LA, a highly homogeneous procedure. Bilateral T8-level ESPB precisely targets the somatic and visceral nociceptive pathways relevant to this surgery, thereby maximizing regional analgesic efficacy (10). Second, our study implemented a higher degree of protocol standardization. We employed a uniform ESPB technique and a consistent multimodal analgesia regimen, minimizing confounding from procedural variability and inconsistent adjuvant analgesia. This rigorous design more accurately isolated the independent opioid-sparing effect of ESPB in this specific pediatric population.

Effective management of acute postoperative pain and opioid-sparing effects may confer substantial benefits for recovery following LA in children (1). First, inadequate control of acute postoperative pain has been consistently associated with an increased risk of persistent postsurgical pain; conversely, robust early pain control may reduce the transition from acute to chronic pain states (40). Second, reduced opioid consumption can attenuate opioid-related adverse effects—such as PONV and gastrointestinal dysfunction—thereby potentially facilitating earlier return of bowel function and oral intake (21, 29, 30). In the present study, we observed numerically lower incidences of PONV (13.5% vs. 23.7%) and pruritus (5.4% vs. 10.5%), as well as shorter times to first flatus (26 vs. 28 h) and first oral intake (29 vs. 31 h) in the ESPB group compared with controls. However, none of these differences reached statistical significance. We attribute these non-significant findings primarily to limited statistical power resulting from our sample size, which was calculated based on the primary outcome (hydromorphone consumption) rather than secondary recovery endpoints. Additionally, the underlying pathophysiology of appendicitis itself—including preoperative inflammation, intraoperative pneumoperitoneum, and manipulation of intra-abdominal structures—may have exerted a dominant influence on gastrointestinal recovery, potentially obscuring the incremental benefits of reduced opioid exposure (41). Future adequately powered trials with recovery-enhanced endpoints as primary outcomes are warranted to definitively evaluate the impact of ESPB on functional recovery following pediatric LA.

ESPB is generally considered a safe regional anesthesia technique in children, owing to its superficial injection site and the ability of ultrasound guidance to clearly visualize and avoid critical structures such as blood vessels, pleura, and neural elements (21, 22, 30). In our study, only two patients (5.4%) experienced a minor procedure-related complication—puncture site bleeding—which resolved completely after 10 min of direct pressure with sterile gauze. No further complications were observed during the postoperative follow-up period. Theoretically, ESPB can be performed with the patient in sitting, lateral decubitus, or prone positions (13, 21). However, as we administered the block after tracheal intubation under general anesthesia, we opted for the prone position, which inevitably added procedural complexity. In contrast, quadratus lumborum block (QLB), another interfascial plane block reported to provide both somatic and visceral analgesia for pediatric abdominal surgery, can be performed with the patient in the supine position (31). However, compared with ESPB, QLB has several limitations: its deeper injection target increases procedural risk and technical difficulty, and its proximity to the lumbar plexus may lead to a higher incidence of postoperative lower limb weakness (11, 13). In contrast, ESPB at the T8 level is relatively superficial, technically straightforward, and carries a lower risk of motor blockade. These characteristics may render ESPB a relatively more attractive option for pediatric LA.

Subgroup analysis demonstrated that the opioid-sparing effect of ESPB was more pronounced in patients with complicated appendicitis than in those with uncomplicated appendicitis, as indicated by a significant group×appendicitis type interaction. This differential effect likely reflects the higher baseline pain intensity and greater opioid requirements associated with complicated appendicitis (24). Importantly, although this subgroup analysis was prespecified in our trial protocol, it should be considered exploratory in nature. The modest sample sizes within each subgroup (*n* = 30 for uncomplicated appendicitis; *n* = 45 for complicated appendicitis) limited the statistical power of the interaction analysis and the precision of the treatment effect estimate. Therefore, these findings must be interpreted with extreme caution, and the enhanced opioid-sparing effect of ESPB in children with complicated appendicitis requires further confirmation in future large-scale, adequately powered multicenter studies.

ESPB has been validated in pediatric open abdominal surgeries (11–13), but its efficacy in laparoscopic procedures like LA remains uncertain. We agree that the pilot study should have verified whether ESPB is effective in pediatric LA and estimated its effect size. Therefore, including an ESPB arm in the pilot study would have been ideal. However, we made a deliberate methodological decision not to include the ESPB arm in our pilot phase due to the extremely limited sample size (*n* = 10). Randomizing such a small cohort into two groups would have yielded highly imprecise effect size estimates with excessively wide confidence intervals, potentially leading to either underpowered or unnecessarily inflated sample size calculations for the definitive trial. Instead, we focused the pilot on assessing procedural feasibility and obtaining baseline data on 24-hour postoperative opioid consumption variability in the control group. These pilot-derived data on outcome variability were then combined with a clinically conservative 30% reduction target, informed by a published high-quality meta-analysis (22), to calculate the formal trial's sample size. We acknowledge that excluding the intervention group from the pilot represents a methodological limitation. However, the main trial demonstrated a 55% reduction in opioid consumption, substantially exceeding the prespecified 30% threshold, which supports the adequacy of our sample size calculation approach and suggests that this design choice did not compromise the validity of our findings.

This study has several limitations. First, we were unable to assess dermatomal sensory blockade coverage after the block or utilize imaging techniques such as three-dimensional CT reconstruction or MRI to visualize local anesthetic spread, which precluded a deeper investigation into the precise analgesic mechanism of ESPB. Second, the study population was restricted to children aged 6–12 years; therefore, the findings may not be directly generalizable to younger children (<6 years) or adolescents (>12 years), in whom anatomical and pharmacokinetic differences may influence block efficacy and safety. Third, the modest sample sizes of the prespecified subgroup analyses limited the statistical power and precision of the test for interaction between appendicitis type and treatment; thus, the subgroup findings cannot be considered confirmatory and require validation in larger, adequately powered studies. Finally, as a single-center trial with a relatively small sample size, the results require validation in larger, multicenter studies to enhance external validity and confirm the robustness of the observed effects.

## 5 Conclusions

In conclusion, a single-injection bilateral ultrasound-guided ESPB at the T8 level, when integrated into a multimodal analgesic regimen with systemic analgesia, significantly reduced opioid consumption during the first 24 postoperative hours and alleviated early postoperative pain in children undergoing LA, without increasing complication rates. These findings support the incorporation of ESPB as a component of multimodal analgesia to optimize postoperative pain management in this population.

## Data Availability

The raw data supporting the conclusions of this article will be made available by the authors, without undue reservation.
