# Supplementary material for: Erector spinae plane block for opioid sparing in children undergoing laparoscopic appendectomy: a randomized controlled trial
Source: Front Pediatr. 2026 May 1;14:1803495. doi: 10.3389/fped.2026.1803495 (PMC13176276; doi:10.3389/fped.2026.1803495)
Supplement: Supplementary file 1 [file Supplementaryfile1.docx]

Supplementary Material 1

# Table 1. Guardian Competency Assessment Checklist for PCIA Operation

| **No.** | **Assessment Item** | **Pass?** |
| --- | --- | --- |
| **1** | **Pain Scale Selection:** Correctly selects the FLACC scale for children < 8 years and the NRS for children ≥ 8 years; demonstrates understanding of scoring criteria. | **☐ Yes ☐ No** |
| **2** | **Threshold Recognition:** Identifies the pain score threshold (score ≥ 4) that authorizes PCIA activation. | **☐ Yes ☐ No** |
| **3** | **Device Identification:** Correctly locates the PCIA pump and identifies the patient-controlled bolus button. | **☐ Yes ☐ No** |
| **4** | **Proper Bolus Technique:** Demonstrates the correct technique for bolus administration (single press, holding for confirmation if required). | **☐ Yes ☐ No** |
| **5** | **Lockout Interval Compliance:** Understands the lockout interval (20 mins) and refrains from repeated button pressing during this period. | **☐ Yes ☐ No** |
| **6** | **Safety Rule:** Strictly adheres to the safety protocol of not administering bolus doses while the child is asleep or excessively sedated. | **☐ Yes ☐ No** |
| **7** | **Alarm Response:** Knows to notify clinical staff immediately upon device alarms rather than ignoring them or attempting self-repair. | **☐ Yes ☐ No** |
| **8** | **Escalation Protocol:** Knows to contact the APS team/nurse for uncontrolled pain (Score ≥ 4 after 20 mins) rather than increasing the frequency of presses. | **☐ Yes ☐ No** |

**Note:** This table presents an English translation of the competency assessment tool; the original Chinese version was utilized for clinical implementation and guardian training in this trial. A guardian was deemed competent only upon successfully passing all 8 items. Consistent with the study protocol, randomization was permitted exclusively for pediatric patients with at least one primary guardian who achieved full competency on this checklist.

**Abbreviations:** PCIA: patient-controlled intravenous analgesia; FLACC: Face, Legs, Activity, Cry, Consolability; NRS: Numeric Rating Scale; APS: Acute Pain Service.
