# Supplementary material for: Erector spinae plane block for opioid sparing in children undergoing laparoscopic appendectomy: a randomized controlled trial
Source: Front Pediatr. 2026 May 1;14:1803495. doi: 10.3389/fped.2026.1803495 (PMC13176276; doi:10.3389/fped.2026.1803495)
Supplement: Supplementary file 2 [file Supplementaryfile2.docx]

Supplementary Material 2

# Table 1. The Analysis Results of the GEE Model for Pain Scores

| **Effect** | **Wald χ²** | **df** | ***P* value** |
| --- | --- | --- | --- |
| **Pain scores at rest** | | | |
| Intercept | 992.21 | 1 | <0.001 |
| Group | 18.01 | 1 | <0.001 |
| Time | 1045.96 | 6 | <0.001 |
| Group × Time | 79.70 | 6 | <0.001 |
| Group × Age group | 0.63 | 2 | 0.729 |
| Group × Time × Age group | 8.23 | 12 | 0.767 |
| **Pain scores during active movement** | | | |
| Intercept | 4040.98 | 1 | <0.001 |
| Group | 11.44 | 1 | <0.001 |
| Time | 1044.18 | 6 | <0.001 |
| Group × Time | 61.28 | 6 | <0.001 |
| Group × Age group | 2.49 | 2 | 0.289 |
| Group × Time × Age group | 9.31 | 12 | 0.676 |

**Note:**

1. Variable coding: group was coded as 0 = Control group and 1 = ESPB group; time was coded as 1 = preoperative, 2 = the highest pain score in the PACU, 3 = 3 hours postoperatively, 4 = 6 hours postoperatively, 5 = 12 hours postoperatively, 6 = 24 hours postoperatively, and 7 = 48 hours postoperatively; age group was coded as 0 = children < 8 years (assessed by FLACC scale) and 1 = children ≥8 years (assessed by NRS).
2. Both resting and active movement pain score models included age group as a covariate, with corresponding interaction terms to adjust for potential bias from age-stratified pain assessment methods.
3. QIC (Quasi-likelihood under the Independence Model Criterion) values: 417.500 for the resting pain model, 454.062 for the active movement pain model.
4. *P* < 0.05 was considered statistically significant.

**Abbreviations:** GEE: generalized estimating equations; df: degrees of freedom; ESPB: erector spinae plane block; PACU: post-anesthesia care unit; FLACC: Face, Legs, Activity, Cry, Consolability; NRS: Numeric Rating Scale.

# Table 2. Estimated Marginal Means of Pain Scores

| **Time** | **Group** | **Emmean** | **SE** | **95%CI Lower** | **95%CI Upper** |
| --- | --- | --- | --- | --- | --- |
| **Pain scores at rest** | | | | | |
| Preoperative | Control group | 3.08 | 0.268 | 2.55 | 3.61 |
|  | ESPB group | 3.16 | 0.28 | 2.61 | 3.71 |
| PACU pain | Control group | 3.92 | 0.202 | 3.52 | 4.31 |
|  | ESPB group | 2.22 | 0.269 | 1.69 | 2.75 |
| Postop 3 h | Control group | 2.79 | 0.204 | 2.39 | 3.19 |
|  | ESPB group | 1.54 | 0.25 | 1.05 | 2.03 |
| Postop 6 h | Control group | 2.61 | 0.19 | 2.23 | 2.98 |
|  | ESPB group | 1.57 | 0.161 | 1.25 | 1.88 |
| Postop 12 h | Control group | 2.47 | 0.129 | 2.22 | 2.73 |
|  | ESPB group | 2.49 | 0.139 | 2.22 | 2.76 |
| Postop 24 h | Control group | 0.66 | 0.178 | 0.31 | 1.01 |
|  | ESPB group | 0.57 | 0.139 | 0.30 | 0.84 |
| Postop 48 h | Control group | 0.61 | 0.115 | 0.38 | 0.83 |
|  | ESPB group | 0.54 | 0.115 | 0.31 | 0.76 |
| **Pain scores during active movement** | | | | | |
| Preoperative | Control group | 5.03 | 0.314 | 4.41 | 5.65 |
|  | ESPB group | 5.13 | 0.397 | 4.35 | 5.91 |
| PACU pain | Control group | 5.76 | 0.216 | 5.34 | 6.19 |
|  | ESPB group | 4.22 | 0.182 | 3.86 | 4.58 |
| Postop 3 h | Control group | 4.71 | 0.216 | 4.29 | 5.13 |
|  | ESPB group | 3.52 | 0.204 | 3.12 | 3.92 |
| Postop 6 h | Control group | 4.66 | 0.214 | 4.24 | 5.08 |
|  | ESPB group | 3.46 | 0.176 | 3.11 | 3.80 |
| Postop 12 h | Control group | 3.47 | 0.112 | 3.25 | 3.69 |
|  | ESPB group | 3.49 | 0.147 | 3.2 | 3.78 |
| Postop 24 h | Control group | 2.55 | 0.155 | 2.25 | 2.86 |
|  | ESPB group | 2.52 | 0.147 | 2.23 | 2.80 |
| Postop 48 h | Control group | 2.55 | 0.111 | 2.33 | 2.77 |
|  | ESPB group | 2.48 | 0.108 | 2.27 | 2.70 |

**Note:** All estimated marginal means were derived from the updated GEE model adjusted for age group as a covariate.

**Abbreviations:** GEE: generalized estimating equations; Emmean: estimated marginal mean; SE: standard error; CI: confidence interval; ESPB: erector spinae plane block.

# **Table 3.** Comparisons of Pain Scores Between Groups

| **Time** | **Comparison** | **ΔEmmean** | **SE** | **95%CI Lower** | **95%CI Upper** | ***P* value** |
| --- | --- | --- | --- | --- | --- | --- |
| **Pain scores at rest** | | | | | | |
| Preoperative | ESPB group vs Control group | -0.08 | 0.41 | -1.50 | 1.34 | >0.999 |
| PACU pain | ESPB group vs Control group | -1.7 | 0.35 | -2.89 | -0.51 | <0.001 |
| Postop 3 h | ESPB group vs Control group | -1.24 | 0.33 | -2.38 | -0.11 | 0.014 |
| Postop 6 h | ESPB group vs Control group | -1.04 | 0.25 | -1.90 | -0.18 | 0.002 |
| Postop 12 h | ESPB group vs Control group | 0.02 | 0.18 | -0.59 | 0.63 | >0.999 |
| Postop 24 h | ESPB group vs Control group | -0.09 | 0.22 | -0.85 | 0.67 | >0.999 |
| Postop 48 h | ESPB group vs Control group | -0.07 | 0.15 | -0.59 | 0.45 | >0.999 |
| **Pain scores during active movement** | | | | | | |
| Preoperative | ESPB group vs Control group | 0.10 | 0.53 | -1.72 | 1.92 | >0.999 |
| PACU pain | ESPB group vs Control group | -1.54 | 0.28 | -2.51 | -0.57 | <0.001 |
| Postop 3 h | ESPB group vs Control group | -1.19 | 0.3 | -2.23 | -0.15 | 0.007 |
| Postop 6 h | ESPB group vs Control group | -1.20 | 0.28 | -2.17 | -0.23 | 0.002 |
| Postop 12 h | ESPB group vs Control group | 0.02 | 0.17 | -0.58 | 0.62 | >0.999 |
| Postop 24 h | ESPB group vs Control group | -0.04 | 0.2 | -0.73 | 0.65 | >0.999 |
| Postop 48 h | ESPB group vs Control group | -0.07 | 0.15 | -0.57 | 0.44 | >0.999 |

**Note:**

1. All pairwise comparisons were derived from the updated GEE model adjusted for age group as a covariate.
2. *P* values were adjusted by the Bonferroni method for multiple comparisons.
3. *P* < 0.05 was considered statistically significant.

**Abbreviations:** GEE: generalized estimating equations; **Δ**Emmean: difference in estimated marginal mean between the ESPB group and the Control group; SE: standard error; CI: confidence interval; ESPB: erector spinae plane block.
