# Supplementary material for: Erector spinae plane block for opioid sparing in children undergoing laparoscopic appendectomy: a randomized controlled trial
Source: Front Pediatr. 2026 May 1;14:1803495. doi: 10.3389/fped.2026.1803495 (PMC13176276; doi:10.3389/fped.2026.1803495)
Supplement: Supplementary file 3 [file Supplementaryfile3.docx]

Supplementary Material 3

# Table 1. Linear regression analysis of 0–24-hour cumulative hydromorphone consumption including ESPB intervention, appendicitis type, and their interaction term

| **Variable** | **B** | **SE** | **95% CI** | ***P* value** |
| --- | --- | --- | --- | --- |
| Intercept | 59.39 | 2.48 | 54.45 to 64.33 | <0.001 |
| Group | | | | |
| ESPB group | -33.15 | 3.40 | -33.91 to -26.39 | <0.001 |
| Appendicitis type | | | | |
| Complicated | 21.39 | 3.12 | 15.17 to 27.61 | <0.001 |
| Interaction (Group × Appendicitis type) | | | | |
| ESPB × Complicated | -9.75 | 4.38 | -18.35 to -1.16 | 0.029 |
| Model summary | | | | |
| Adjusted *R²* | 0.846 | | |  |
| *F* statistic | 136.66 | | | <0.001 |
| Degrees of freedom | 3, 71 | | |  |

**Note:**

1. Dependent variable: 0–24-hour cumulative hydromorphone consumption (μg/kg).
2. Reference categories: Control group (0 = Control group, 1 = ESPB group); Uncomplicated appendicitis (0 = Uncomplicated, 1 = Complicated).
3. Adjusted *R²* = 0.846 indicates the model explains 84.6% of variance in hydromorphone consumption.
4. The interaction term (ESPB × Complicated, B = -9.75, *P* = 0.029) indicates ESPB provided an additional 9.75 μg/kg reduction in 0–24-hour hydromorphone consumption in patients with complicated appendicitis, compared with those with uncomplicated appendicitis.
5. *P* < 0.05 was considered statistically significant.

**Abbreviations:** B: unstandardized regression coefficient; SE: standard error; CI: confidence interval; ESPB: erector spinae plane block.
